# A Calcineurin Regulator MoRCN1 Is Important for Asexual Development, Stress Response, and Plant Infection of *Magnaporthe oryzae*

**DOI:** 10.3389/fpls.2022.925645

**Published:** 2022-06-16

**Authors:** Caiyun Liu, Tiangu Liu, Ziwei Lv, Mengyuan Qin, Zhiguang Qu, Ziwei Zhang, Fuyan Li, Deng Chen, Xinrong Zhang, Xiao-Lin Chen, Mi Shen

**Affiliations:** ^1^Hubei Key Laboratory of Economic Forest Germplasm Improvement and Resources Comprehensive Utilization, College of Biology and Agricultural Resources, Huanggang Normal University, Huanggang, China; ^2^State Key Laboratory of Agricultural Microbiology and Provincial Key Laboratory of Plant Pathology of Hubei Province, College of Plant Science and Technology, Huazhong Agricultural University, Wuhan, China

**Keywords:** calcipressin, calcineurin, calcium signaling, fungal infection, *Magnaporthe oryzae*

## Abstract

The calcium/calcineurin signaling pathway plays a key role in the development and virulence of plant pathogenic fungi, but the regulation of this signaling pathway is still not clear. In this study, we identified a calcineurin regulator MoRCN1 in the plant pathogenic fungus *Magnaporthe oryzae* and found it is important for virulence by regulating the calcineurin pathway. *MoRCN1* deletion mutants were severely decreased in colony growth and conidia formation. More importantly, the deletion of *MoRCN1* led to a significant reduction in virulence due to defects in appressorium formation and invasive growth. The Δ*Morcn1* mutants were more sensitive to different stresses and induced host ROS accumulation, suggesting a role of *MoRCN1* in stress adaptation. We found that MoRCN1 directly interacted with the calcineurin catalytic subunit MoCNA and affected its protein stability, which was therefore important for regulating the calcineurin pathway. Transcriptome analysis showed that MoRCN1 significantly activated 491 genes and suppressed 337 genes in response to calcium ion, partially overlapped with the MoCRZ1-bound genes. Gene Ontology and KEGG pathway analyses indicated that MoRCN1-regulated genes were enriched in stress adaptation, lipid metabolism, and secondary metabolite biosynthesis, reflecting a function of MoRCN1 in host cell adaptation. Altogether, these results suggest MoRCN1 functions as a regulator of the calcium/calcineurin signaling pathway for fungal development and infection of host cells.

## Introduction

The calcium/calcineurin signaling pathway is widely conserved in eukaryotic cells, which is activated by the G-protein coupled receptors when induced by external stimuli (Steinbach et al., 2007). Plasma membrane phospholipase C is induced for hydrolyzing phosphatidyl inositol-1,4-bisphosphate (PIP2) to diacylglycerol (DAG) and inositol 1,4,5-triphosphate (IP3). Subsequently, IP3 activates releasing of calcium to the cytosol from the intracellular stores, when the calcium ions bind to calmodulin to activate the calcineurin, a serine/threonine protein phosphatase (Steinbach et al., 2007). Calcineurin is a heterodimer composed of two subunits, namely, the catalytic subunit A (calcineurin A, CNA) and a regulatory subunit B (calcineurin B, CNB) (Dickman and Yarden, 1999). Calcineurin is known to be involved in a myriad of cellular processes and signal transduction pathways (Klee et al., 1998; Rusnak and Mertz, 2000), such as T cell activation (Ferreira et al., 1993), long-term memory (Mansuy et al., 1998), apoptosis in neurons, and skeletal and cardiac muscle growth and differentiation (Rusnak and Mertz, 2000). As a conserved protein phosphatase from yeast to humans, the function of calcineurin is to dephosphorylate the downstream substrate targets, which regulates functions of the targets, for example, the capacity of nuclear translocation (Crabtree, 2001). Cyclosporin A (CsA) and tacrolimus (FK506) can effectively inhibit the biological activity of calcineurin, making targeting calcineurin a promising antifungal drug development strategy (Juvvadi et al., 2014a).

Since calcineurin is involved in regulating various signal pathways, it is conceivable that several endogenous regulatory factors have been identified (Pinchai et al., 2009; Juvvadi et al., 2015). In yeast and mammals, there is a family of conserved proteins called “regulators of calcineurin (RCANs)” (also called calcipressins) (Davies et al., 2007), which directly bind to the catalytic subunit of calcineurin to regulate the phosphorylase activity of calcineurin. The DSCR1 has been reported as the key factor in inhibiting calcineurin causing Down syndrome in humans (Fuentes et al., 2000; Arron et al., 2006). In the yeast *Saccharomyces cerevisiae*, deletion of *RCN1* (a homolog of human DSCR1) caused sensitivity to cation stress (Kingsbury and Cunningham, 2000). In *Aspergillus fumigatus*, the deletion of the *cbpA* gene (a homolog of *RCN1*) resulted in reduced hyphal growth and attenuated virulence. Additionally, the Δ*cbpA* strain displayed improved Ca^2+^ tolerance compared to the wild-type (WT) strain under high-calcium-level conditions (Pinchai et al., 2009). In *Cryptococcus neoformans*, the *cbp1* mutant strains exhibit modest defects in growth under stress conditions and in virulence (Görlach et al., 2000; Fox and Heitman, 2005). However, the roles of calcineurin regulator in plant pathogens remains largely unknown.

It remains controversial how the RCN1 protein modulates calcineurin function precisely. Domain analysis of RCN1 revealed the presence of a conserved serine-proline repeat (SP repeat; FxISPPxSPP motif), which could be phosphorylated at two serine residues by mitogen-activated kinase (MAPK), followed by glycogen synthase kinase 3 (GSK3β) (Vega et al., 2002). The regulation of calcineurin by RCNs is involved in the phosphorylation level of RCNs, which causes stimulation of calcineurin (Hilioti et al., 2004), and autoregulatory negative-feedback loop of calcineurin pathway causing inhibition of calcineurin signaling (Kishi et al., 2007). It is still necessary to further determine how RCN1 regulates calcineurin.

*Magnaporthe oryzae* is a destructive plant pathogenic fungus, leading to rice blast disease (Zhang et al., 2016). During the infection process, on the host surface, *M. oryzae* forms a melanized appressorium, which facilitates its breaching of the host cuticle by forming a penetration peg. Then, in the host cells, *M. oryzae* spreads through invasive hyphae from cell to cell (Ebbole, 2007; Wilson and Talbot, 2009; Zhang et al., 2019). The calcium signaling pathway has been shown to be important for the development, stress response, and virulence of *M. oryzae* (Nguyen et al., 2008; Choi et al., 2009a,b; Kim et al., 2010). MoCNA, the catalytic subunit of calcineurin in *M. oryzae*, plays important roles in signal transduction pathways involved in infection-related morphogenesis and pathogenicity (Nguyen et al., 2008; Choi et al., 2009a). MoCRZ1, a C2H2 transcription factor which is activated by calcineurin dephosphorylation, is required for conidiation and infection by affecting functional appressorium formation and interaction with host cells (Choi et al., 2009b). MoCRZ1 directly binds to many infection-related genes and regulates their expression (Kim et al., 2010). In this study, we identified a calcineurin regulator *MoRCN1* in *M. oryzae* and found that it is required for asexual development, stress response, and virulence to the host plant in *M. oryzae*. *MoRCN1* responds to calcium signals and directly binds to the calcineurin MoCNA to maintain its protein stability. Transcriptome analysis showed that, upon calcium ion treatment, MoRCN1 activated 491 genes and suppressed 337 genes, some of which were Crz1-regulated genes. Functional classification indicated that RCN1-regulated genes were involved in many processes, including stress adaptation, lipid metabolism, and secondary metabolite biosynthesis, which demonstrated a detailed regulation of MoRCN1 in invasive growth.

## Experimental Procedures

### Strains and Culture Conditions

In this study, *M. oryzae* strain P131 was regarded as a wild type, and all the fungal strains were cultured on oatmeal tomato agar plates at 28°C to assess growth and conidiation capacity. To test various stress sensitivity, all strains were grown on complement medium (CM) agar added with 0.1 mg/ml CFW (Sigma-Aldrich, USA), 0.2 mg/ml Congo Red (Sigma-Aldrich), 0.005% sodium dodecyl sulfate (SDS), 0.5 M NaCl, or 10 mM H_2_O_2_. Then the colony diameters were measured after 5 days of growth (Liu et al., 2018). For calcium sensitivity assessment, the strains were cultured on minimum medium (MM) supplemented with 200 mM CaCl_2_ or/and 0.2 ng ml^−1^ FK506 for 5 days. Genome DNA and total RNA were extracted from mycelium after 36 h of culture in CM (180 rpm).

### Phylogenetic Tree Analysis and Protein Alignment

The protein sequence of MoRCN1 was regarded as a query to blast homolog proteins from other different species on the Enzembl Fungi website (http://fungi.ensembl.org). Clustal_W was used to align the amino acid sequences of homologous proteins in the species (Larkin et al., 2007). The phylogenetic tree was accomplished using MEGA7, and the percentage of replicate in which associated taxa clustered together in the bootstrap test (1,000 replicates) was shown next to the branches (Kumar et al., 2016).

### Gene Disruption and Complementation of *MoRCN1*

For gene deletion vector construction, 1.5-kb upstream and downstream fragments of *MoRCN1* were separately amplified from the genome DNA of P131 and then cloned into the deletion vector pKNH (Supplementary Table 6) (Chen et al., 2014). The constructed deletion vector pKNH-RCN1 was transformed into protoplasts of P131 to gain the *MoRCN1* deletion transformants. For gene complementation assay, a fragment containing a 1.5-kb native promoter region, coding region, and 0.5-kb terminator region of MoRCN1 was cloned to the pKN vector (Supplementary Table 6) (Chen et al., 2014). The constructed vector pKN-RCN1 was transformed into protoplasts of the Δ*Morcn1* mutant, and subsequent transformants were selected by 400 μg/ml neomycin (Amresco, Solon, OH, USA) and confirmed by the PCR-mediated method. All the primers used in this study are shown in Supplementary Table 7 (see Supporting Information).

### Virulence Test and Infection Process Observation

For virulence test on rice, conidia suspensions with a concentration of 5 × 10^4^ conidia/ml in 0.025% Tween 20 were sprayed onto 21-day-old rice leaves (*Oryza sativa* cv. LTH). The rice samples were kept in a growth chamber at 28°C with 90% humidity in dark for 36 h, then exposed to light. Lesion formation could be observed at 5 days after incubation. To observe the infection process, 7-day-old barley (*Hordeum vulgare* cv. E9) were inoculated with conidia suspension (3 × 10^4^ conidia/ml) of different strains, and the infection process was observed at different time points using a Nikon Ni90 microscope (Nikon, Tokyo, Japan).

### Quantitative Real-Time PCR Analysis

Total RNA samples were extracted from CM media cultivating for 36 h (28°C, 160 rpm) using TRIzol reagent (Invitrogen, Carlsbad, CA, USA) and were treated with DNaseI to digest genome DNA. A total of 1 mg RNA was used to synthesis cDNA, which was used for the template of qRT-PCR to evaluate the gene expression level. The qRT-PCR experiment was performed by using an SYBR Green PCR Master Mix kit (Takara, Dalian, China) on an ABI 7500 real-time PCR system (Applied Biosystems, Foster City, CA, USA).

### Co-Immunoprecipitation (Co-IP) Assay

To confirm the interaction between MoRCN1 and MoCNA, the coding region of *MoRCN1* was cloned into vector pKN-Flag to gain pKNFlag-RCN1, and *MoCNA* was cloned into pRGTN (containing GFP) to gain pRGTN-CNA. The pKNFlag-RCN1 and pRGTN-CNA were co-transformed into the WT strain, and the resulting transformants were selected by GFP signal detection and western blot with an anti-FLAG antibody. To perform the Co-IP experiment, the total number of proteins of the selected transformants were extracted and incubated with 20 μl of anti-FLAG (DYKDDDDK) magnetic beads magarose beads (B26101, Bimake, Beijing, China) for 4 h at 4°C. The magnetic beads were washed three times using TBS buffer (50 mM Tris, 0.15 M NaCl, and pH7.4), and proteins were eluted from the anti-FLAG beads and analyzed by western blot with the anti-GFP antibody. The pKNFlag-RCN1 and pRGTN-CNA vectors were also, respectively, transformed into the WT strain and used as controls and analyzed by western blot.

### Yeast Two-Hybrid Assays

The full-length cDNA of *MoRCN1* was cloned into pGBKT7 as the bait construct pGBKT7-RCN1, and the cDNA of MoCNA was cloned into pGADT7 as the prey construct pGADT7-CNA. The pGBKT7-RCN1 and pGADT7-CNA were co-transformed into the yeast strain AH109 according to the manufacturer's instructions (Clontech, San Francisco, CA, USA). All transformants were assayed with 1 × 10^6^ cells/μl droplet on SD-Leu-Trp-His and SD-Leu-Trp-His with 20 mg/ml X-α-gal plates. Self-activation showed that both pGBKT7-RCN1 and pGADT7-CNA had no self-activation. The interaction of pGBKT7-53 and pGADT7-T was used as a positive control, and the interaction of pGBKT7-Lam and pGADT7-T was used as a negative control.

### Staining Assays

The DAB (3,3-diaminobenzidine, Sigma-Aldrich, USA) solution with a concentration of 0.1 mg/ml was used to detect ROS in host cells infected by different strains. For CFW staining, mycelia and conidia were harvested from CM medium and stained with 10 μg/ml CFW (Sigma-Aldrich, United States) for 10 min in the dark, then observed under the Nikon microscope Ni90 (Nikon, Tokyo, Japan). Glycogen was detected by KI/I_2_ staining solution (60 mg/ml KI and 10 mg/ml I_2_) (Thines et al., 2000). Lipid droplets were stained using Nile Red solution, consisting of 50 mM Tris/maleate buffer (pH 7.5), 20 mg/ml polyvinylpyrrolidone, and 2.5 mg/ml Nile Red (Sigma-Aldrich, St. Louis, MO, USA). For different kinds of staining assays, the spore suspension (5 × 10^5^ spores/ml) was dropped on plastic coverslips and kept in growth chamber and stained for 15 min at different time points, then detected using an epifluorescence microscope (Ni90, Nikon, Tokyo, Japan).

### Subcellular Localization

The MoRCN1:GFP fusion vector was generated by cloning the MoRCN1 promoter and coding region into the N-terminus of GFP in the vector pGTN (Supplementary Table 6). The resulting pGTN-RCN1 vector was transformed into the Δ*Morcn1* mutant, and the positive transformants were used to observe subcellular localization at different developmental stages and infection processes using a confocal microscope Leica TCS SP8 (Leica Microsystems, Germany).

### RNAseq and Bioinformatics Analysis

The wild-type strain P131 and the Δ*Morcn1* mutants were incubated for 48 h in CM medium with or without 200 mM CaCl_2_ (WT, WT[Ca^2+^], Δ*Morcn1*, Δ*Morcn1*[Ca^2+^]). Three biological replicates were used for each sample. Total RNA from each mycelium sample was extracted using Trizol reagent (Invitrogen, Carlsbad, CA, USA). The mRNA was enriched and purified using poly-T oligo-attached magnetic beads and fragmented into small pieces using divalent cations. The RNA fragment was then used to synthesize cDNA, “A” base was added, and adapter was connected. After purification and enrichment by PCR amplification, the samples were used to obtain the final cDNA library for sequencing using combined probe anchor synthesis sequencing methods (Beijing Genomics Institution, Beijing, China).

Differentially expressed genes were analyzed by the DESeq function in the DESeq2 package, and expression with log2|FC| > 1 with <0.05 *p*-adjust values was defined as DEGs. Gene Ontology enrichment analysis was performed using AgriGO toolkits (Du et al., 2010) using p-adjust value cutoffs (0.05) for significance. KEGG enrichment of metabolic pathways was analyzed using the R package clusterProfilter version 3.16 (Yu et al., 2012). Pathview (Version 3.11) was used to produce detailed maps for the selected pathways (Luo et al., 2017).

## Results

### Identification of *MoRCN1* in *M. oryzae*

Using the *S. cerevisiae* RCN1 protein sequence as a query to perform a BLASTP search in the *Magnaporthe* genome database (Ensembl Fungi), the *M. oryzae* MoRCN1 (MGG_03218) was identified. Multiple protein sequence alignments indicated that *M. oryzae* MoRCN1 protein sequence shares high homology with numerous proteins of different eukaryotes, including *S. cerevisiae, Fusarium oxysporium, C. neoformans, Danio rerio*, and *Homo sapiens* (Supplementary Figure 1A). The *M. oryzae* MoRCN1 protein is predicted to be composed of 266 amino acid residues that contain a calcipressin domain (Supplementary Figure 1B). Phylogenetic tree analysis was analyzed using MEGA7 software, which showed that the MoRCN1 protein shares the highest homology with ascomycete fungi, including *Neorospora crassa, Fusarium oxysporum, F. graminearum*, while is not closely related to basidiomycete fungi *Ustilago maydis* and *C. neoformans* (Supplementary Figure 1C).

### Expression Patterns of *MoRCN1* During Different Developmental Stages

To gain insight into the functions of *MoRCN1* of *M. oryzae*, we first examined its expression pattern at developmental stages, including in mycelium, conidium, germ tube, appressoria, and invasive hypha at 18, 24, and 48 h post inoculation (hpi). According to the qRT-PCR result, in comparison to the hyphal stage, the expression of *MoRCN1* was highly expressed in conidium, appressorium, primary infection hypha, and late infection hypha (Supplementary Figure 2), suggesting it plays important roles in these stages.

To further determine the functional roles of *MoRCN1* in *M. oryzae*, we subsequently disrupted this gene in the wild-type strain P131 by using a homologous recombination strategy (Supplementary Figure 3A). After a PCR-based screening (Supplementary Figure 3B), two independent *MoRCN1* deletion mutants (KO-1 and KO-2) were obtained. We also performed a complementation experiment by introducing the native promoter-driven MoRCN1 coding region into KO-1. Most of the complementation transformants were recovered to the wild type in growth, conidiation, and virulence, and one of which, cRCN1, was chosen for further analysis.

### Deletion of *MoRCN1* Affects Fungal Vegetative Growth and Conidial Formation

**Figure 1:**
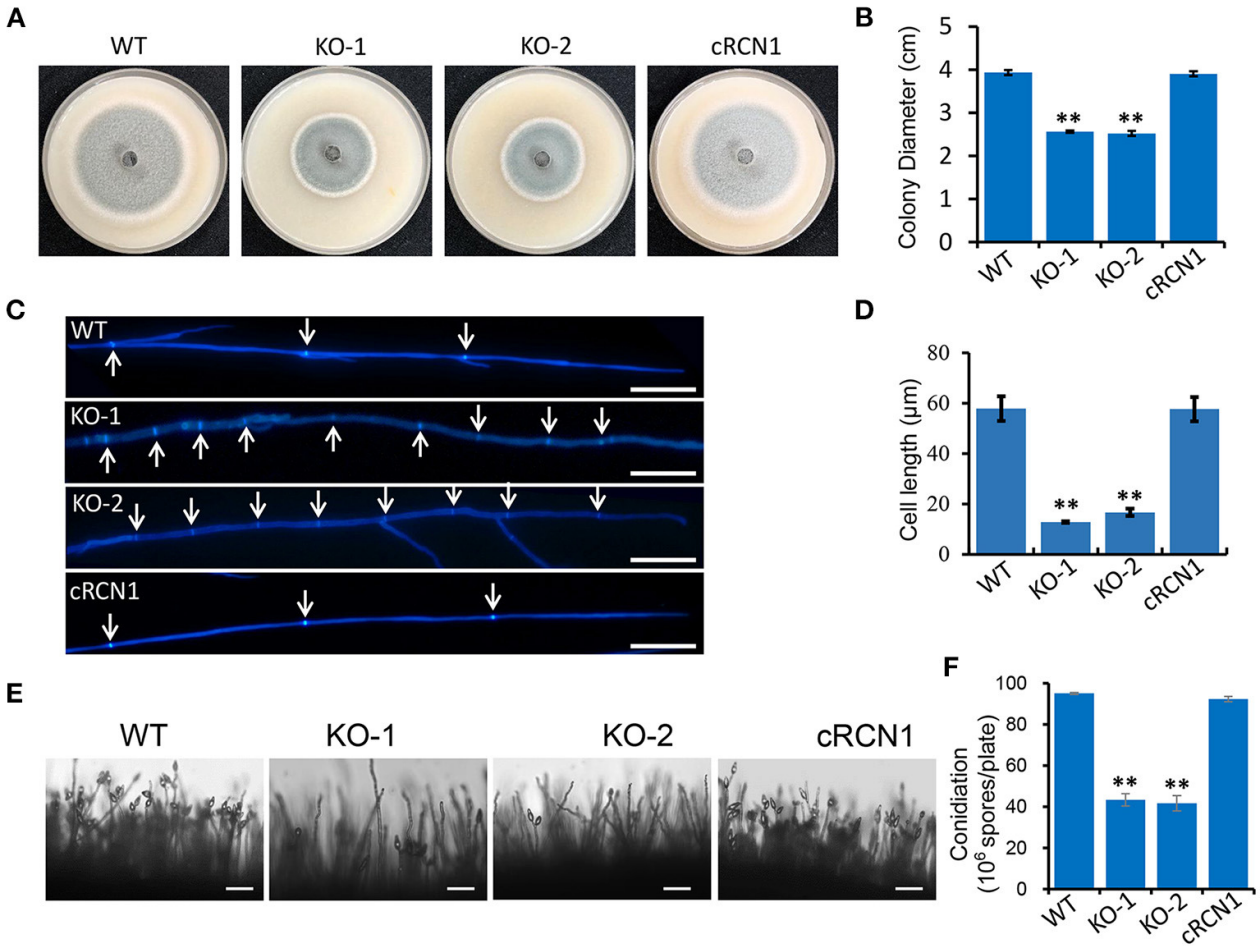
MoRCN1 is involved in vegetative growth. **(A)** Colony morphology of the wild-type P131, Δ*Morcn1* mutants, and complemented strains were observed on oatmeal tomato agar (OTA) plates at 28°C for 5 days. **(B)** The colony diameters were measured and subjected to statistical analysis. Error bars represent standard deviation, and asterisk represents significant differences (*P* < 0.01). **(C)** Hyphal tips of the wild-type P131, Δ*Morcn1* mutants, and complemented strains. The arrows indicate septa between cells. Bar 20 μm. **(D)** Cell length of the apical and subapical cells in the hyphae tips of strains in **(C)**. Means ± SD were calculated from three independent experiments. Significant differences compared to the wild type (WT) are indicated by an asterisk (*P* < 0.01). **(E)** Conidial and conidiophore formation were observed under a light microscope. The indicated strains were grown on OTA plates for 5 days. Bar 50 μm. **(F)** Statistical analysis of conidiation of the wild type, Δ*Morcn1* mutants, and complemented strains. Error bars represent the standard deviation, and asterisks represent significant differences (*P* < 0.01).

To investigate the roles of MoRCN1 in vegetative growth, we observed the colony growth and mycelial morphology of the Δ*Morcn1* mutants. Compared with that of the wild type, we found a severe growth reduction in the Δ*Morcn1* mutants, since the colony diameter of the Δ*Morcn1* mutants was around 1.6 cm shorter than that the wild type after cultivating on OTA plates for 120 h at 28°C (Figures 1A,B). Staining of mycelial tip cells with Calcofluor White showed that Δ*Morcn1* appeared to form shorter hyphal compartments than the wild type (Figures 1C,D).

We also tested whether the deletion of *MoRCN1* affected the conidia development. We found that the Δ*Morcn1* mutants formed sparse conidia on the conidiophores when observed under the microscope. In contrast, the wild-type and complement strains formed dense conidia on the conidiophores (Figure 1E). Coordinately, the Δ*Morcn1* mutants formed only 42% of the wild-type conidia on OTA plates (Figure 1F). These results showed that *MoRCN1* is required for asexual growth and conidia formation.

### Deletion of *MoRCN1* Led to Attenuated Virulence

**Figure 2:**
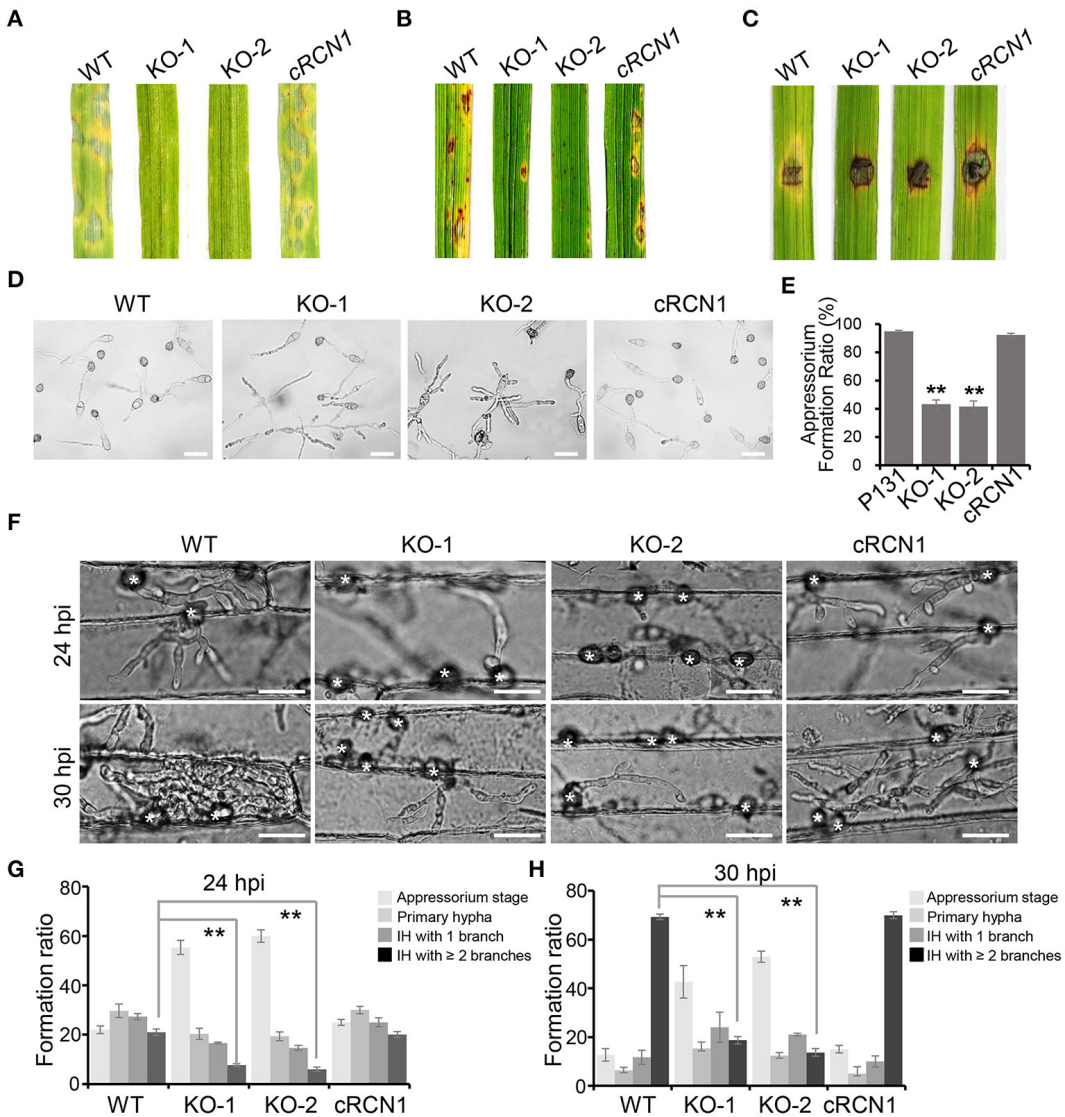
Deletion mutants of *MoRCN1* lead to severe reduction in virulence. **(A)** Virulence test on barley leaves. Lesions formed on barley leaves were sprayed by conidia (3 × 10^4^ conidia/ml) of indicated strains and observed at 5-day post inoculation (dpi). **(B)** Virulence test on rice seedlings. Lesions formed on rice leaves were induced by spray inoculation with conidia (1.5 × 10^5^ conidia/ml) of indicated strains and observed at 5 dpi. **(C)** Lesions formed on wounded rice leaves. The wounded rice leaves were inoculated by mycelia blocks of different strains and observed at 4 dpi. **(D)** Appressorium formation were observed under a light microscope. The conidia of tested strains were inoculated on hydrophobic surfaces and calculated at 18 hpi. Bar 30 μm. **(E)** Statistical analysis of appressoria formation ratio. Error bars represent the standard deviation, and asterisks represent significant differences among strains (*P* < 0.01). **(F)** Invasive hyphae (IH) formed in barley epidermal cells infected by different strains at 24 and 30 hpi. Bar = 30 μm. **(G)** Formation ratio of different patterns of infection structures at 24 hpi. **(H)** Formation ratio of different patterns of infection structures at 30 hpi. For **(G)** and **(H)**, percentages of appressoria (AP), primary invasive hyphae (PH), and IH with branches in barley epidermal cells were calculated. Means and standard deviation were calculated from three independent replicates. Significant differences compared to the WT are indicated by asterisks (*P* < 0.01).

To test whether disruption of *MoRCN1* affected virulence of *M. oryzae*, the barely leaves and rice seedlings were sprayed with conidia from different strains. A severe reduction in virulence was observed in the Δ*Morcn1* mutant-infected plants compared with those infected by the wild type (Figures 2A,B). Δ*Morcn1* mutants are also cannot spread well on the wounded rice leaves, suggesting a defect in host cell invasion (Figure 2C). We then observed whether deletion of *MoRCN1* affected appressorium formation. On hydrophobic surface, more than 90% of conidia in the wild type formed appressoria at 12 hpi, but only 43% of conidia in the Δ*rcn1* mutants formed appressoria, indicating that *MoRCN1* plays a role in appressorium formation (Figures 2D,E). To further determine why the Δ*Morcn1* mutants were attenuated in virulence, we observed the cellular infection processes in barley epidermis cells. At 25 hpi, around 60% of the Δ*Morcn1* mutant conidia still remained at the appressorium stage and 20% formed primary invasive hyphae, while 75% of the wild-type conidia formed invasive hyphae and 45% formed more than two branches. At 30 hpi, nearly 70% of the wild-type IH formed more than two branches, whereas it was no more than 20% in the Δ*Morcn1* mutants (Figures 2F–H). Taken together, *MoRCN1* is indispensable for appressorium formation, penetration, and invasive growth in the host cells.

### *MoRCN1* Is Required for Utilization of Glycogen and Lipid Metabolism During Appressorium Formation

**Figure 3:**
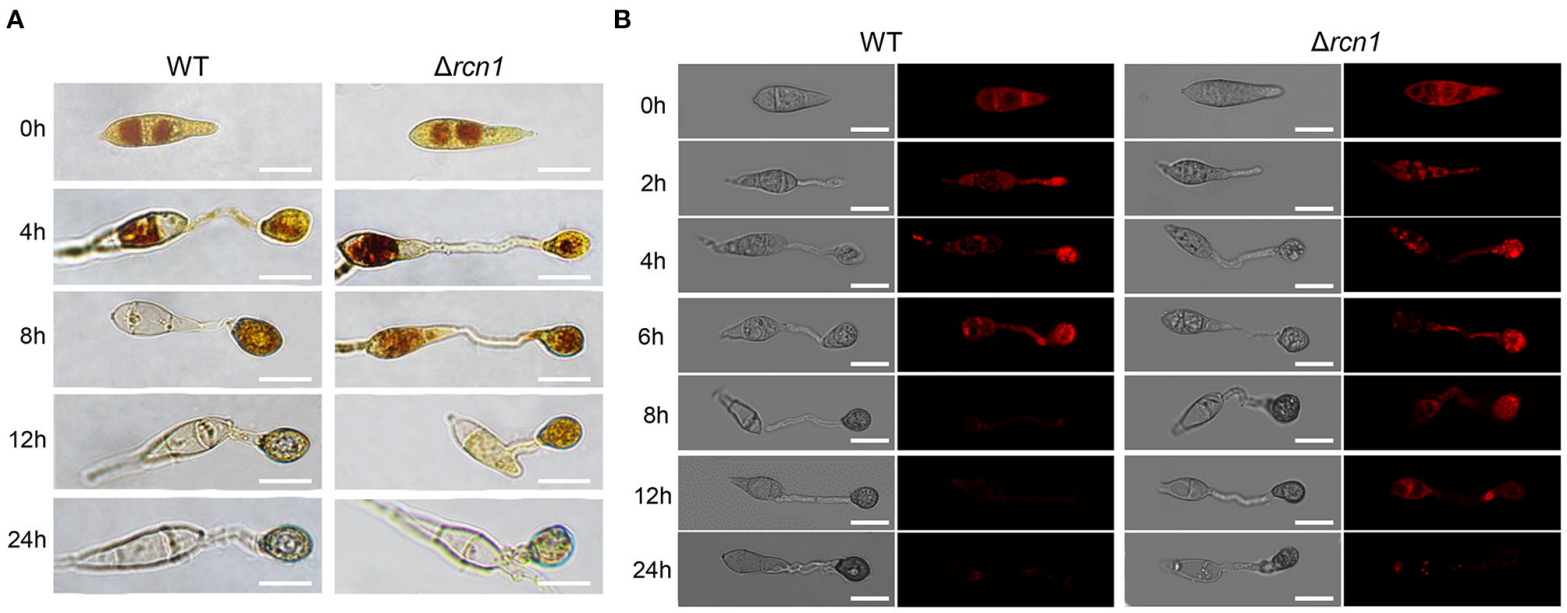
Deletion of *MoRCN1* delays the utilization of glycogen and lipid droplets during appressorium formation. **(A)** Glycogen utilization staining assay. P131 and Δ*Morcn1* mutants were stained with KI/I_2_ solution at different time points, and yellowish-brown glycogen deposits became visible and were observed using microscopy. Bar 10 μm. **(B)** Lipid droplet degradation staining assay. Lipid bodies of conidia were stained with Nile Red at different time points. Bar 10 μm.

Since the formation of a functional appressorium is accompanied by the degradation of fatty acids and the utilization of glycogen, we next detected the cellular distribution of glycogen and lipids during the appressorium development. Glycogen can be stained with I_2_/KI solution, while the lipid droplets can be detected by Nile Red staining. In wild type, the glycogen was translocated from conidia to nascent appressoria, and rapidly consumed after 12 hpi, and disappeared at 24 hpi. While in the Δ*Morcn1* mutants, the glycogen of the Δ*Morcn1 mutants* is utilized much slower and were still noticeable at 24 hpi (Figure 3A). Similarly, in wild type, lipid droplets were transferred from conidia to nascent appressoria and were consumed after 8 hpi, while in the Δ*Morcn1* mutants, degradation of the lipid droplets was much slower, and a large amount of lipid droplets could be still detected at 24 h (Figure 3B). These results indicated that deletion of *MoRCN1* affects glycogen and lipid metabolism, which is important for conidia storage utilization and functional appressorium formation for host penetration.

### The Δ*Morcn1* Caused Accumulation of Reactive Oxygen Species (ROS) in Host Cells

**Figure 4:**
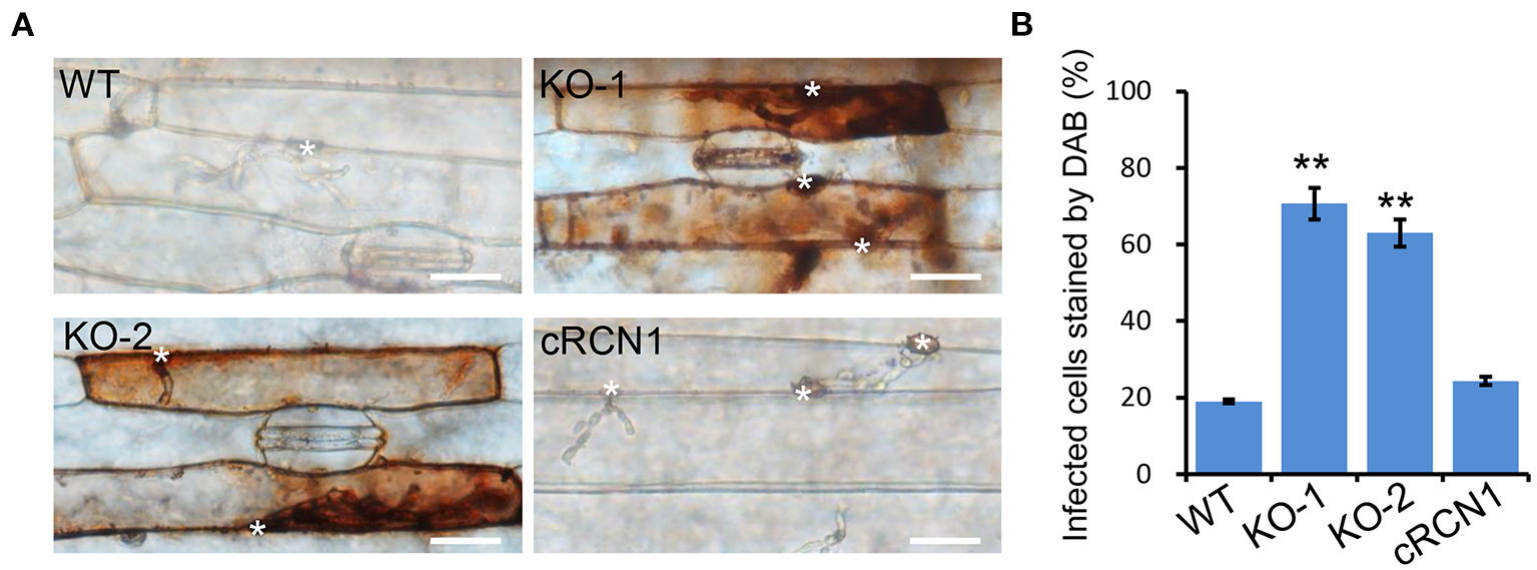
Deletion mutants of *MoRCN1* induce accumulation of host reactive oxygen species (ROS). **(A)** DAB staining assay. Barley epidermis infected by the wild type, Δ*Morcn1* mutants, and complemented strains was stained with 3,3-diaminobenzidine (DAB) at 30 hpi. The asterisk indicates the appressorium. Bar 20 μm. **(B)** Statistical analysis for percentage of the infected cells stained by DAB. Significant differences were indicated by asterisks (*P* < 0.01).

Retardation in the invasive growth of *M. oryzae* is usually caused by failure to suppress host ROS accumulation. To test whether Δ*Morcn1* mutants could induce host ROS accumulation, the *M. oryzae*-infected barley epidermis cells at 30 hpi were stained with 3, 3-diaminobenzidine (DAB). Approximately 70% of the Δ*Morcn1* mutant-infected host cells had ROS accumulation with reddish-brown color, while no more than 30% of the wild-type strain infected host cells were detected, confirming that the Δ*Morcn1* mutants were impaired in the detoxification of ROS produced by the host cells (Figures 4A,B). This result indicated that *MoRCN1* is important for suppressing host defense response, especially the oxidative stresses.

### *MoRCN1* Is Important for Stress Response

**Figure 5:**
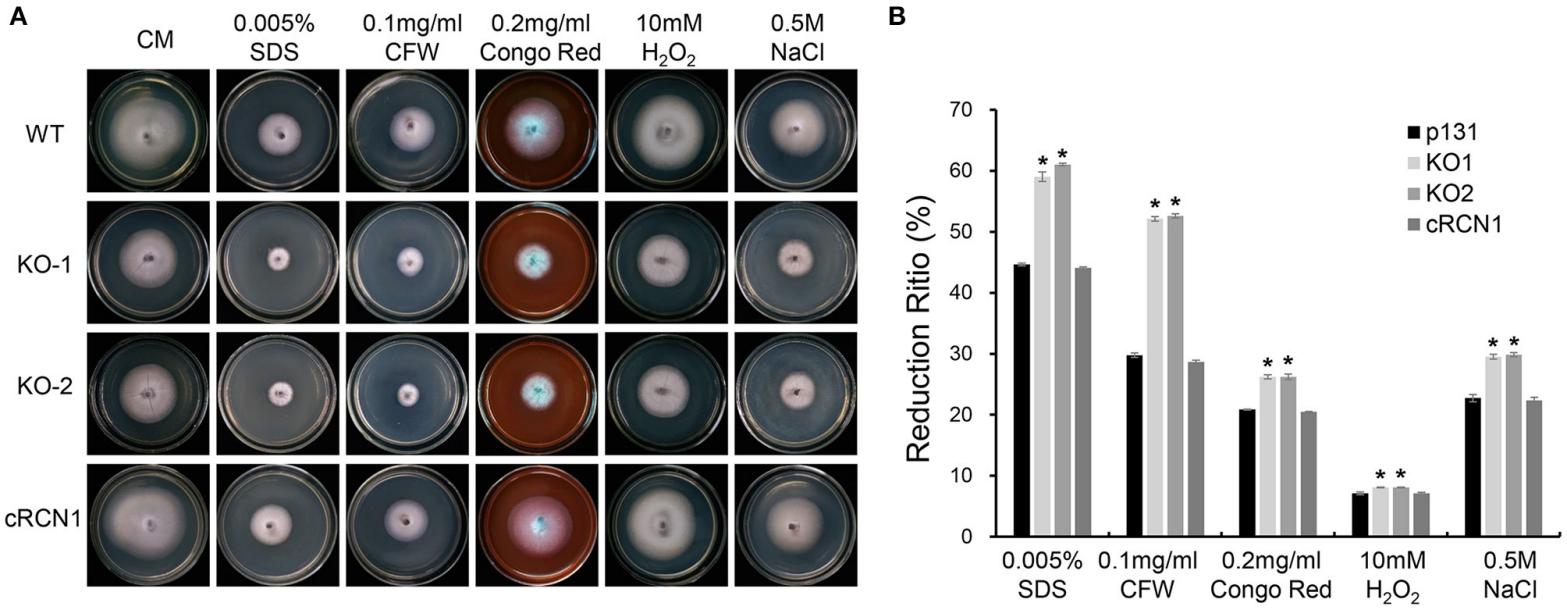
Deletion mutants of *MoRCN1* are sensitive to cell wall disturbing agents, oxidative stress, and osmotic stress. **(A)** Colony morphology of the wild type, Δ*Morcn1* mutants, and complemented strain on CM plates supplemented with different cell wall disturbing agents, 10 mM H_2_O_2_ and 0.5 M NaCl agents. The colonies were photographed at 5 dpi. **(B)** Statistical analysis of growth reduction rates of colony growth under different stress agents. Means and standard errors were calculated from three independent replicates. Significant differences were indicated by asterisks (*P* < 0.05).

To further determine the role of MoRCN1 in stress response, we tested the sensitivities of the Δ*Morcn1* mutants to different cellular stress agents. The tested strains were cultured on CM plates amended with different cellular stress agents, including those involved in cell wall disruption (0.2 mg/ml CR, 0.1 mg/ml Calcofluor White [CFW], and 0.005% SDS), osmotic stress (0.5 M NaCl), and oxidative stress (10 mM H_2_O_2_). The result showed that Δ*Morcn1* mutants were more sensitive to all the above mentioned stresses (Figures 5A,B), suggesting that *MoRCN1* plays a central role in responding to different kinds of stresses, including cell wall integrity and osmotic and oxidative stress responses.

### *MoRCN1* Is Localized in the Cytoplasm and Accumulated in the Nucleus Upon Calcium Induction

**Figure 6:**
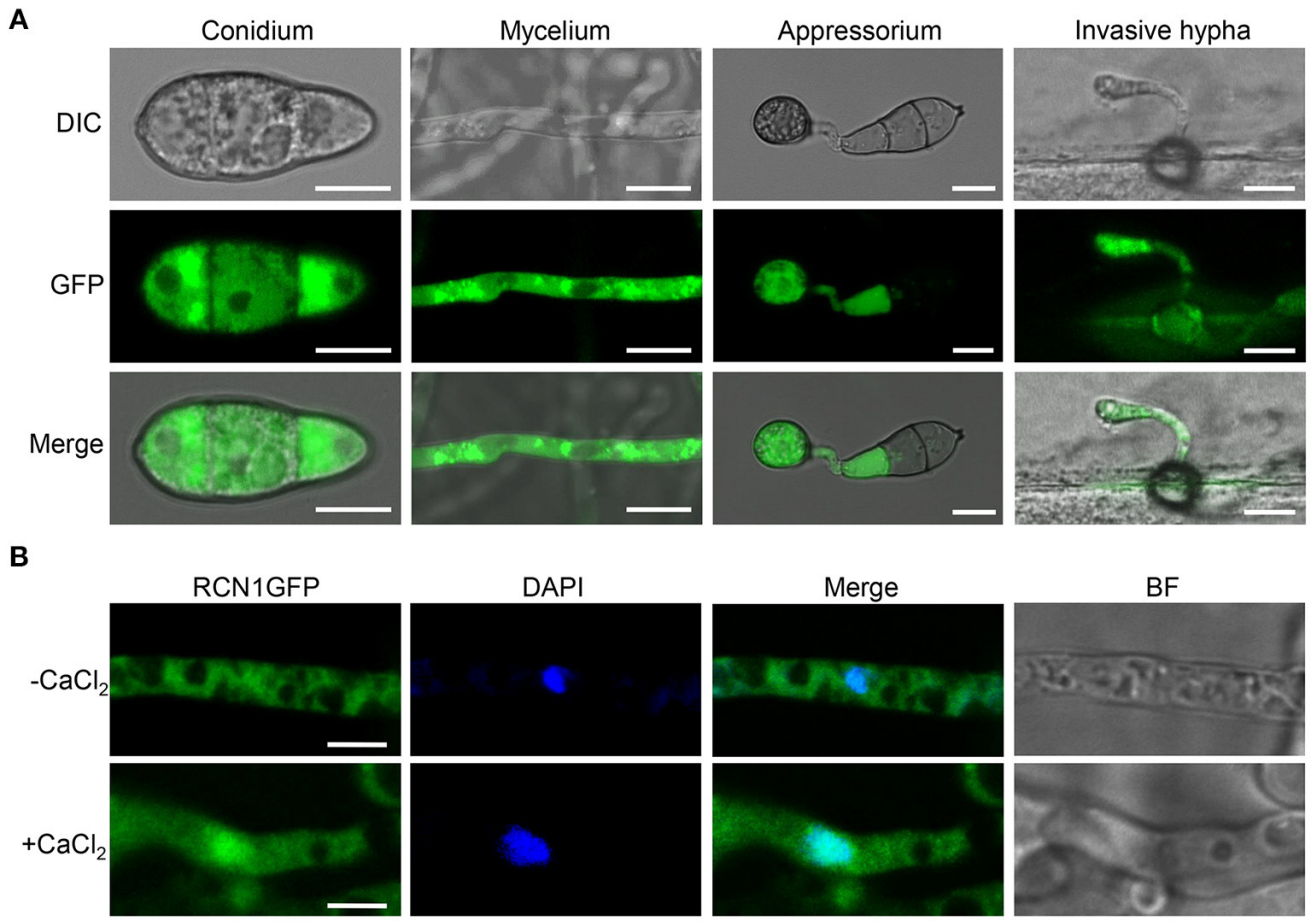
Subcellular localization of MoRCN1 at different developmental stages of *M. oryzae*. **(A)** Subcellular localization of GFP-Rcn1 in conidia (CO), mycelium (MY), appressoria (AP), and primary invasive hyphae (PH). Bar 10 μm. **(B)** Subcellular localization of GFP-Rcn1 in mycelium with and without CaCl_2_ (200 mM) induction. Nuclei were visualized by using the fluorescent dye DAPI. Bar 10 μm.

To study the subcellular localization of MoRCN1, the GFP was fused with the C-terminus of MoRCN1 protein and transformed into Δ*Morcn1* mutants. The transformants were all recovered to the wild type, and one of which was used for subcellular observation. Through the fluorescence microscopy, we observed that MoRCN1-GFP was distributed in the cytoplasm at the stages of conidia, vegetative hyphae, appressorium, and invasive hyphae, with a stronger fluorescence accumulation around the nuclei (Figure 6A). Since high concentration of calcium can activate the calcium signaling pathway and induce calcineurin to dephosphorylate target proteins in eukaryotes (Park et al., 2019), we wonder whether calcium ions could also induce nuclear localization of MoRCN1. As expected, we observed MoRCN1-GFP was evidently translocated into the nuclei under 200 mM CaCl_2_ treatment in vegetative hyphae (Figure 6B). This result showed that MoRCN1 was located in the cytoplasm under normal conditions, and a calcium ion could promote its transfer into the nucleus, suggesting a role of *MoRCN1* in calcium signaling response.

### *MoRCN1* Binds to MoCNA and Affect Its Protein Stability

**Figure 7:**
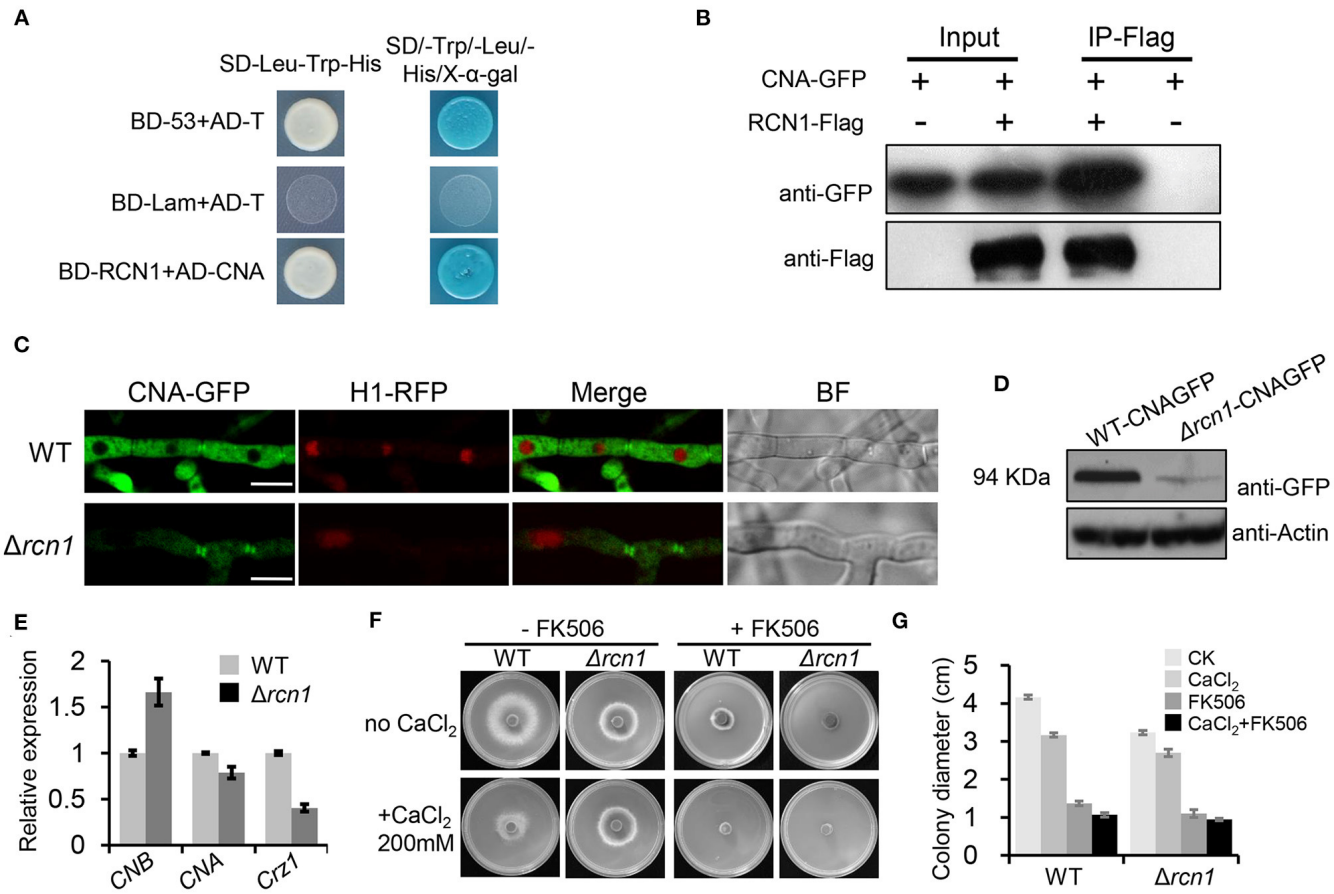
MoRCN1 interacts with MoCNA and affects its protein level. **(A)** Yeast-two-hybrid assay for the interaction between MoRCN1 and MoCNA. Yeast transformants expressing the prey and bait constructs were assayed for growth on SD-Leu-Trp and SD-Leu-Trp-His-Ade plates stained with X-α-gal. The positive control was the interaction between pGBKT7-53 (BD-53) and pGADT7-T (AD-T), and the negative control was the interaction between pGBKT7-Lam (BD-Lam) and pGADT7-T. **(B)** Co-immunoprecipitation (CoIP) analyses. The 3 × flag-RCN1 and GFP-MoCNA were co-expressed in the wild-type strain. The Co-IP experiment was performed with the anti-GFP beads, and the isolated protein was analyzed by western blot using anti-FLAG and anti-GFP antibodies. **(C)** Subcellular localization of MoCNA in WT and Δ*Morcn1* mutants. MoCNA and H1-RFP were co-transformed into WT and Δ*Morcn1* mutants. The co-localization was observed under the confocal. The arrows point to the mycelial septum. Bar 10 μm. **(D)** The protein content of MoCNA in WT and Δ*Morcn1* mutants was detected by western blot. **(E)** The expression level of MoCNA, MoCNB, and MoCRZ1 in WT or the Δ*Morcn1* mutants. *MoAct1* was used as an internal control. The expression level of different genes in WT was set as 1. **(F)** Growth of different strains in the presence of CaCl_2_. WT and Δ*Morcn1* mutants were inoculated onto MM plates containing CaCl_2_ (200 mM) with or without the calcineurin inhibitor FK506 (1 ng/ml). The colony was photographed at 120 h. **(G)** The colony diameters of WT and Δ*Morcn1* mutants in **(F)**.

MoRCN1 has been reported to be a calcipressin that regulates calcineurin by directly binding in *Botrytis cinerea, Aspergillus fumigatus*, and *C. neoformans* (Görlach et al., 2000; Pinchai et al., 2009; Harren et al., 2012). To experimentally prove whether MoRCN1 interacts with the calcineurin subunit A (MoCNA) in *M. oryzae*, we performed a yeast two hybrid (Y2H) assay. As expected, MoCNA clearly interacted with its putative regulator MoRCN1 *in vitro* (Figure 7A).

To further confirm the interaction of MoRCN1 and MoCNA *in vivo*, we performed a co-immunoprecipitation (CoIP) assay. We generated the MoRCN1-3× FLAG and MoCNA-GFP constructs, which were co-transformed into the wild-type strain. As a control, the two vectors were also, respectively, transformed into the wild-type strain. The MoRCN1-interacted proteins were enriched from the total protein with anti-FLAG magnetic beads, and the eluted protein from the beads was detected with GFP antibody. As shown in Figure 7B, the MoRCN1-FLAG fusion protein co-immunoprecipitated with the MoCNA. Thus, MoRCN1 also interacts with MoCNA *in vivo*.

In the wild-type strain, MoCNA-GFP was clearly located in the cytoplasm and the septum but not in the nucleus. In the Δ*Morcn1* mutants, the localization of MoCNA-GFP was not changed, but its fluorescence intensity was dramatically decreased compared with that in the wild type (Figure 7C). Under the treatment of 200 mM CaCl_2_, the localization of MoCNA-GFP was not changed either (Supplementary Figure 4). Subsequent western blot analysis also confirmed a significant reduction in the protein level of MoCNA-GFP in the Δ*Morcn1* mutants (Figure 7D). The qRT-PCR experiment demonstrated only a slight reduction in expression of *MoCNA* in Δ*Morcn1* mutants compared with the wild type, suggesting that the reduction in the protein level should be due to a decrease in its transcription level (Figure 7E). We also detected an increased expression level of *MoCNB*, the regulatory subunit of calcineurin, in the Δ*Morcn1* mutants (Figure 7E), suggesting a compensation effect resulted by protein reduction in the calcineurin. Together, these results indicated that MoRCN1-regulated protein stability of the calcineurin regulatory subunit MoCNA in *M. oryzae*.

### The *MoRCN1* Deletion Mutants Displayed Improved Calcium Tolerance

To further investigate whether *MoRCN1* plays a role in the calcineurin pathway, we compared the growth of wild-type and Δ*Morcn1* strains on minimal medium under high-calcium levels (200 mM CaCl_2_) with or without the calcineurin inhibitor FK506. Interestingly, the Δ*Morcn1* strain exhibited a greater hyphal growth than the wild-type strain in the medium containing CaCl_2_ (Figures 7F,G). However, in the presence of FK506, the increased calcium tolerance of the Δ*Morcn1* strain was completely reversed, indicating that *MoRCN1* contributed to calcium tolerance in a calcineurin-dependent manner (Figures 7F,G), suggesting that *MoRCN1* may play a negative feedback regulatory role in response to calcium tolerance.

### *MoRCN1* Deletion Alters Expression Pattern of Genes Under the High Calcium Conditions

**Figure 8:**
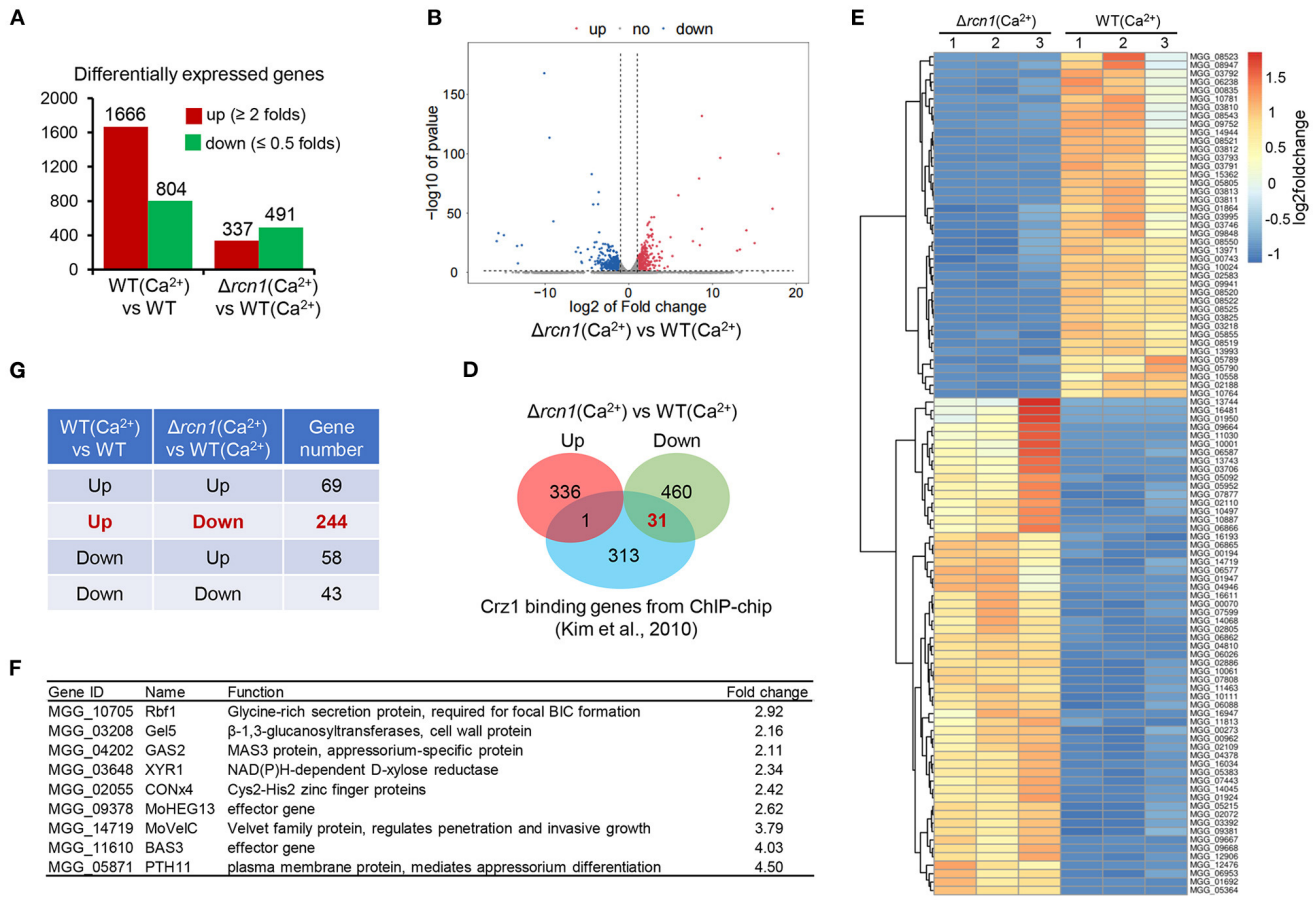
Integrative analysis of RNA-seq genes affected by calcium in WT and Δ*Morcn1* mutants. **(A)** Differentially expressed genes of different comparing pairs obtained from RNAseq data. **(B)** Expression fold changes of genes in Δ*Morcn1* induced by Ca^2+^ compared with WT induced by Ca^2+^ (|log2[FC]| > 1; *p*-adj <0.05). **(C)** Gene numbers shared in different comparing pairs of upregulated or downregulated genes in **(A)**. **(D)** Overlap of MoRCN1-regulated and Crz1-regulated genes reported by Kim et al. (2010). **(E)** Heatmap shows hierarchical clustering of genes from transcriptome data. WT (Ca^2+^), the wild type strain induced by CaCl_2_; Δ*Morcn1* (Ca^2+^), the Δ*Morcn1* mutants induced by CaCl_2_. **(F)** Previously reported pathogenesis genes in differentially expressed genes of Δ*Morcn1*(Ca^2+^) compared with WT(Ca^2+^).

To understand the impact of high Ca^2+^ concentration on gene expression at the transcriptional level in the wild-type and Δ*Morcn1* strains, we performed a comparative transcriptomic analysis in vegetative hyphae treated with 200 mM CaCl_2_ for 1 h. In WT, compared with that under the normal condition, the expression of 1,666 genes was increased under the calcium ion-treatment condition, while that of 804 genes was decreased (Figure 8A). In Δ*Morcn1* mutants, under the calcium ion-treatment condition, we identified 337 upregulated genes and 491 downregulated genes compared with that of the wild-type strains (Figures 8A,B; Supplementary Figure 5). This result indicates that MoRCN1 plays both activator and repressor roles in gene expression, and relatively more genes are activated by MoRCN1. Interestingly, around one half of the downregulated genes in Δ*Morcn1* mutants were also upregulated genes in the wild-type strain induced by calcium ions (Figure 8C), indicating that MoRCN1-mediated calcium response accounts for a large part of the calcium response of *M. oryzae*.

We also compared MoRCN1-regulated genes with the MoCRZ1-binding genes, which was reported by Kim et al. (2010). MoCRZ1 is an important calcium-responsive transcription factor in *M. oryzae* (Kim et al., 2010). As shown in Figure 8D, 31 downregulated genes of Δ*Morcn1* were also listed in the MoCRZ1-binding genes, but only one gene was listed in the upregulated genes of Δ*Morcn1*, suggesting that MoRCN1 plays an important role in activating MoCRZ1, the C2H2 transcription factor activated by calcineurin dephosphorylation (Kim et al., 2010).

Hierarchical clustering of MoRCN1-regulated genes gives an overview of calcium induction and highlights major clusters (Figure 8E). We also searched the previously published genes in the list of MoRCN1-regulated genes and found some upregulated genes in Δ*Morcn1*, including Rbf1, Gel5, RAS2, XYR1, CONx4, MoHEX13, MoVelC, BAS3, and PTH11. Among them, *RAS2* and *PTH11* are known as appressorium formation regulators (DeZwaan et al., 1999; Park et al., 2006). *MoVelC* and *CONx4* are involved in development of conidial morphology (Kim et al., 2014; Cao et al., 2016). MoHEX13 and BAS3 are effector genes that function in invasive growth (Mosquera et al., 2009). *RBF1* is a virulence gene that is essential for the focal BIC formation during invasive growth (Nishimura et al., 2016) (Figure 8F). These data suggest that MoRCN1 could negatively regulate different genes involved in development and infection upon high calcium induction.

### Classification of MoRCN1-Regulated Genes Induced by Calcium

**Figure 9:**
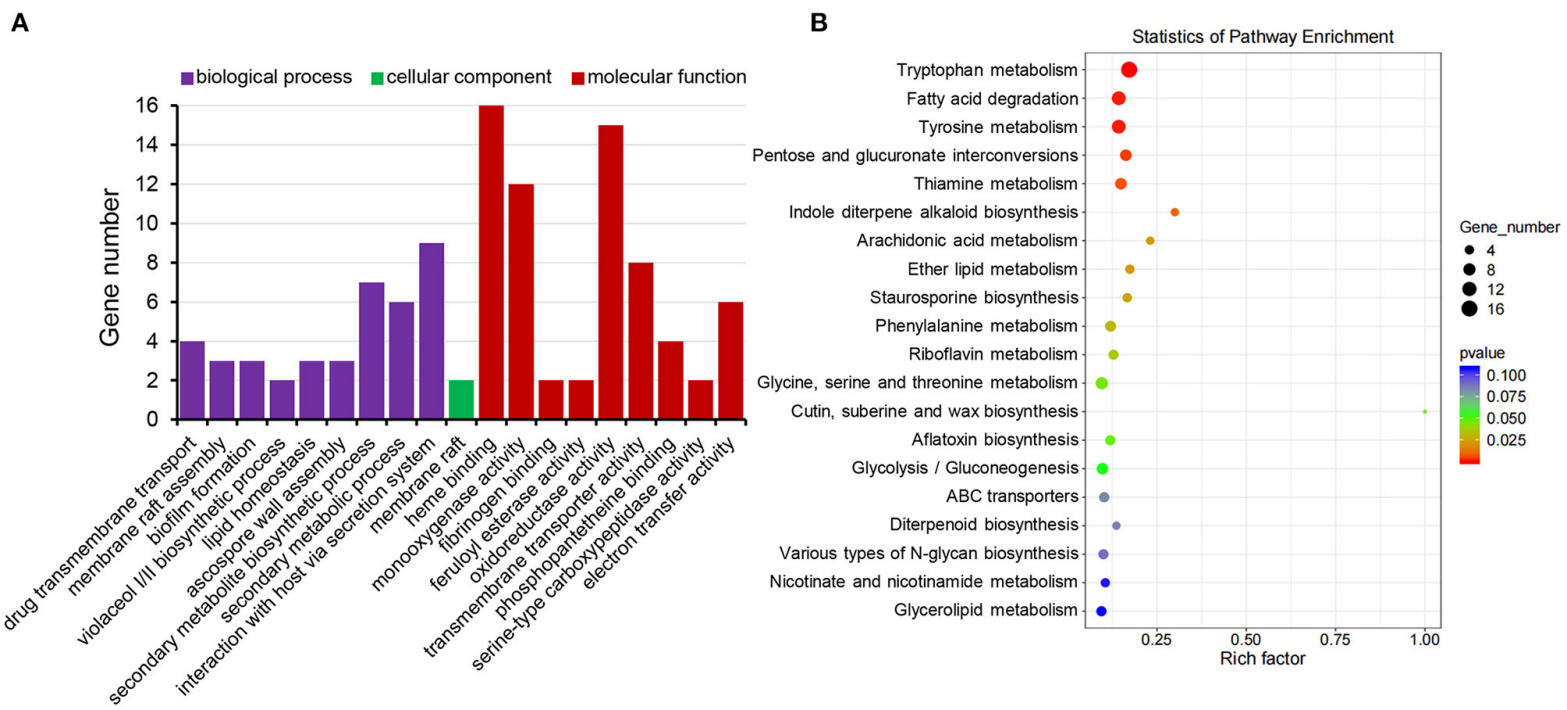
Functional classification of MoRCN1 regulated genes relevant to Ca^2+^ induction. **(A)** Gene Ontology terms of biological processes, cellular components, and molecular functions enriched in MoRCN1 regulated genes related to Ca^2+^ induction. **(B)** KEGG analysis of enriched in MoRCN1 regulated genes related to Ca^2+^ induction.

Gene Ontology (GO) enrichment analysis of differentially expressed genes showed that biological processes of interaction with the host *via* secretary system, secondary metabolic process, secondary metabolite biosynthesis, ascospore wall assembly, lipid homeostasis, membrane raft assembly, as well as transmembrane transport were significantly enriched, most of which were related to stress response. For cellular components, the membrane raft was enriched. For molecular function, heme binding, monooxygenase activity, oxidoreductase activity, and electron transfer activity were enriched (Figure 9A), most of which were related to energy metabolism. In sum, results of the GO enrichment analysis were consistent with the fact that the MoRCN1 is involved in response to different stresses.

Kyoto Encyclopedia of Genes and Genomes (KEGG) pathway enrichment analysis shows that the MoRCN1-regulated genes induced by calcium were mainly involved in pathways related to amino acid metabolisms (tryptophan metabolism, tyrosine metabolism, phenylalanine metabolism, and glycine, serine, and threonine metabolism), lipid metabolism (fatty acid degradation, ether lipid metabolism, and glycerolipid metabolism), secondary metabolite metabolism (thiamine metabolism, indole diterpene alkaloid biosynthesis, diterpenoid biosynthesis, arachidonic acid metabolism, riboflavin metabolism, nicotinate and nicotinamide metabolism, staurosporine biosynthesis, and aflatoxin biosynthesis), and ABC transporters (Figure 9B). These results suggest that *MoRCN1* is important for metabolism pathways for invasive growth.

## Discussion

The calcium/calcineurin signaling pathway has been reported to be important for the development and virulence of the plant pathogenic fungi (Nguyen et al., 2008; Choi et al., 2009a,b; Kim et al., 2010), but the regulatory mechanism of this signaling pathway is still not very clear. In this study, we identified a calcineurin regulator MoRCN1 in *M. oryzae* and found that it is important for asexual development, stress response, and infection process. More importantly, we provide the evidence that MoRCN1 regulates calcineurin signaling pathway by affecting protein stability of the calcineurin catalytic subunit MoCNA. Transcriptome data suggested important roles of *MoRCN1* in stress response and adaptation to host cells.

Calcineurin has been reported to be required for mycelial growth, conidiation, appressorium formation, and pathogenicity in *M. oryzae* (Choi et al., 2009b). In this study, MoRCN1 is also required for these development and infection processes as a calcineurin regulator. Deletion of *MoRCN1* resulted in a severe reduction in hyphae growth, sporulation, and virulence (Figures 1, 2), consistent with CbpA previously reported in the human pathogen *A. fumigatus* (Pinchai et al., 2009) and the plant gray mold pathogen *B. cinerea* (Harren et al., 2012). The virulence reduction in the Δ*Morcn1* mutants should be attributed to reduced appressorium formation ratio, decreased penetration, and blocked invasive growth. The utilization of spore storage of the Δ*Morcn1* mutants were significantly retarded, resulting in a decreased penetration (Figures 3A,B). In plant pathogenic fungi, as appressorium formation and maturation process are usually regulated by the cAMP signaling pathway (Li et al., 2019, 2020), MoRCN1 may be also involved in this signaling pathway. In fact, the calcium/calcineurin signaling pathway has been widely reported to be related to cAMP signaling pathway in fungi (Bencina et al., 2005; Chen et al., 2021). It is interesting to determine the roles of MoRCN1 in cAMP signaling pathway in the future.

*MoRCN1* plays a key role in stress responses that facilitate the invasive growth of *M. oryzae*. Deletion of *MoRCN1* in *M. oryzae* resulted in sensitivity to all tested stresses, including cell wall perturbing reagents, salt stress, osmotic stress, and oxidative stress (Figures 5A,B). The Δ*Morcn1* mutants also resulted in an accumulation of host ROS, the first line of defense for host cells against pathogens. It is evident that MoRCN1-mediated stress adaptation can counteract with different kinds of stresses in host cells, not only ROS, which is important for survival and invasive growth of *M. oryzae*. This possibility is also reflected by studies of the calcium/calcineurin signaling pathway in many other fungi such as *S. cerevisiae, C. albicans, C. neoformans*, and *B. cinerea* (Odom et al., 1997; Stathopoulos and Cyert, 1997; Yoshimoto et al., 2002; Bader et al., 2003; Onyewu et al., 2004; Santos and de Larrinoa, 2005; Karababa et al., 2006; Harren et al., 2012), which demonstrated some other stresses such as pH values and high temperature. Our transcriptome data also suggest that a large number of stress adaptation-related genes were regulated by MoRCN1, including genes involved in secondary metabolic process, secondary metabolites biosynthesis (such as thiamine metabolism, riboflavin metabolism, aflatoxin biosynthesis, and diterpenoid biosynthesis), ABC transporters, and genes with monooxygenase activity, oxidoreductase activity, and electron transfer activity requiring an oxidative mechanism. How calcineurin is well regulated in the cell has not been well understood in fungi. We showed MoRCN1-GFP was mainly located in the cytoplasm in normal conditions and during different developmental stages, but accumulated in the nuclei when induced with 200 mM CaCl_2_ for 1 h, suggesting MoRCN1 in nuclei is closely related to the calcium/calcineurin signaling pathway. A similar phenomenon is also found in the gray mold *B. cinerea*, where BcRcn1 is localized basically in the cytoplasm but also has a stronger fluorescence around or in the nuclei under stress conditions, such as 20 mM CaCl_2_ or alkaline pH (Harren et al., 2012). Regulators of calcineurin (RCANs) have been determined to inhibit and stimulate calcineurin signaling in fungi and mammals through binding to the catalytic subunit of the phosphatase directly (Kingsbury and Cunningham, 2000; Horner et al., 2006). In *A. fumigatus*, CnaA-EGFP complexing with CnaB is localized at the septa and is required for normal calcineurin function at the hyphal septum (Juvvadi et al., 2014b). In this study, we demonstrate that MoCNA has functions in septum formation (Figure 7C) and that MoRCN1 has no influence on the septum distribution of MoCNA. In addition, Figure 1C shows that Δ*Morcn1* mutants can form normal septa. The distribution of MoCNA was not changed in Δ*Morcn1* mutants, suggesting that MoRCN1 does not affect subcellular localization of MoCNA. Interestingly, although the protein level of MoCNA was severely decreased in Δ*Morcn1* mutants, transcription of *MoCNA* was not significantly reduced. It may be due to the compensation effect that expression of the calcineurin regulatory subunit *MoCNB* was evidently elevated. Therefore, MoRCN1 can maintain protein stability of the calcineurin catalytic subunit MoCNA to regulate calcium/calcineurin signaling pathway. Studies have also shown that the loss of RCANs is accompanied by the loss of calcineurin (Shin et al., 2006). However, it is not clear that whether this protein stability regulation is direct or indirect. Other studies have reported that the phosphorylation of RCN1 stimulates the phosphatase activity of calcineurin (Kishi et al., 2007), therefore, besides protein stability regulation, it is also possible that MoRCN1 can regulate phosphatase activity in *M. oryzae*.

Transcriptome data suggested that MoRCN1 is one of the downstream regulators of the calcium signaling pathway, but not the sole one. We also noticed that although some MoRCN1-regulated genes upon calcium treatment also MoCRZ1-bound genes, the number was limited. This phenomenon suggested that the transcription factor MoCRZ1 is not the only target of MoRCN1-mediated calcium/calcineurin signaling pathway, and some other targets must be functional upon calcium signaling response.

FK506 and cyclosporine A are widely used as specific inhibitors of calcineurin to augment the potency of azole class antifungal drugs (Marchetti et al., 2000). However, some assays show that these drugs cause immunosuppression and unwanted effects on other tissues (Liu et al., 1991; Rothermel et al., 2000). It is necessary to explore endogenous inhibitors of calcineurin as part of future drug targeting strategies. A better understanding of how calcineurin is regulated in living cells, for example, MoRCN1, is beneficial for developing more selective ways for developing potential fungicides.

## Data Availability Statement

The original contributions presented in the study are publicly available. This data can be found here: National Center for Biotechnology Information (NCBI) BioProject database under accession number PRJNA797545 (https://www.ncbi.nlm.nih.gov/bioproject/PRJNA797545).

## Author Contributions

MS and X-LC conceived the study, designed the experiments, and supervised the project. CL, TL, ZL, MQ, ZQ, ZZ, FL, DC, and XZ performed the experiments. X-LC, CL, and MS analyzed data and wrote the manuscript. All authors discussed the results, contributed to the final manuscript, and contributed to the article and approved the submitted version.

## Funding

This study was supported by the National Natural Science Foundation of China (Grants 31871909 and 32072365) and the Fundamental Research Funds for the Central Universities (2021ZKPY007).

## Conflict of Interest

The authors declare that the research was conducted in the absence of any commercial or financial relationships that could be construed as a potential conflict of interest.

## Publisher's Note

All claims expressed in this article are solely those of the authors and do not necessarily represent those of their affiliated organizations, or those of the publisher, the editors and the reviewers. Any product that may be evaluated in this article, or claim that may be made by its manufacturer, is not guaranteed or endorsed by the publisher.
