# Supplementary material for: A Calcineurin Regulator MoRCN1 Is Important for Asexual Development, Stress Response, and Plant Infection of Magnaporthe oryzae
Source: Front Plant Sci. 2022 Jun 16;13:925645. doi: 10.3389/fpls.2022.925645 (PMC9244802; doi:10.3389/fpls.2022.925645)
Supplement: Supplementary file 1 [file Data_Sheet_1.pdf]

Supplemental Figures

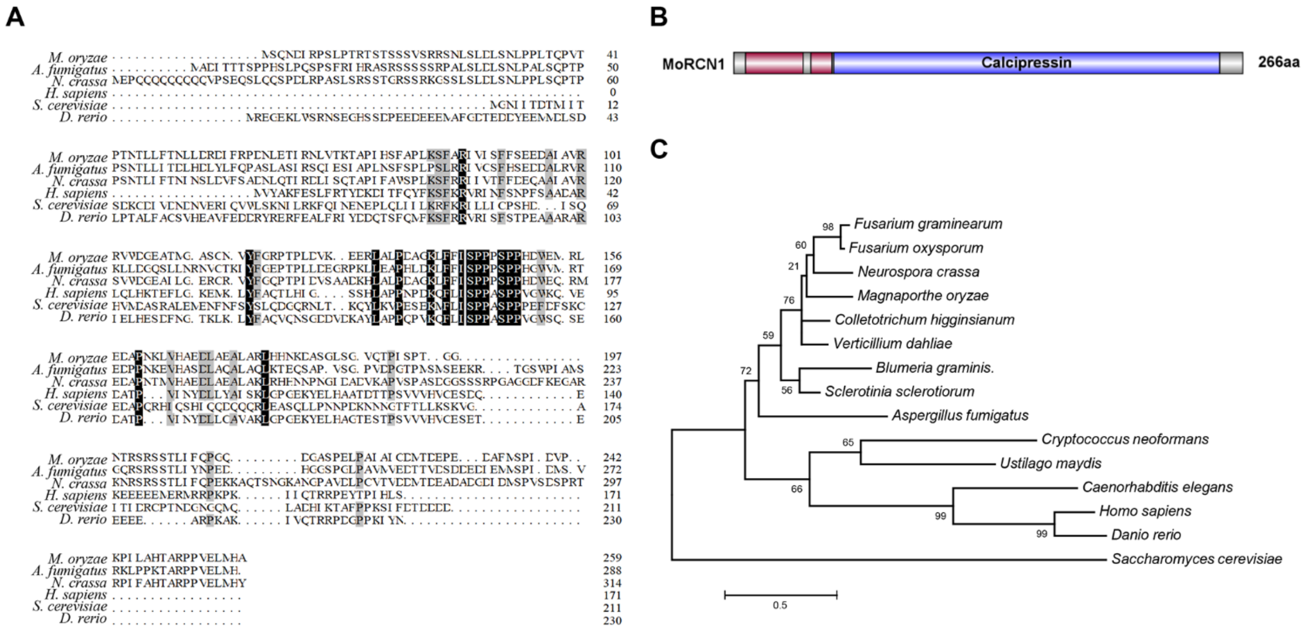

Supplementary Figure 1 | Analyses of RCN1 functional domain and systematic evolution. **(A)** Alignment of the amino acid sequences of LDP1 from *M. oryzae* and other selected model organisms. Amino acid sequences were aligned with ClustalW (http://www.ch.embnet.org/software/ClustalW.html). Identical residues are colored by black regions, and similar residues are colored by gray regions. **(B)** Analysis of MoRCN1 protein structure. Region marked blue shows Calcipressin domain. **(C)** A phylogenetic tree of the RCN1 amino acid sequences in different species. Numbers at nodes indicate the percentages of occurrence. Scale bar indicates the number of amino acid differences per site.

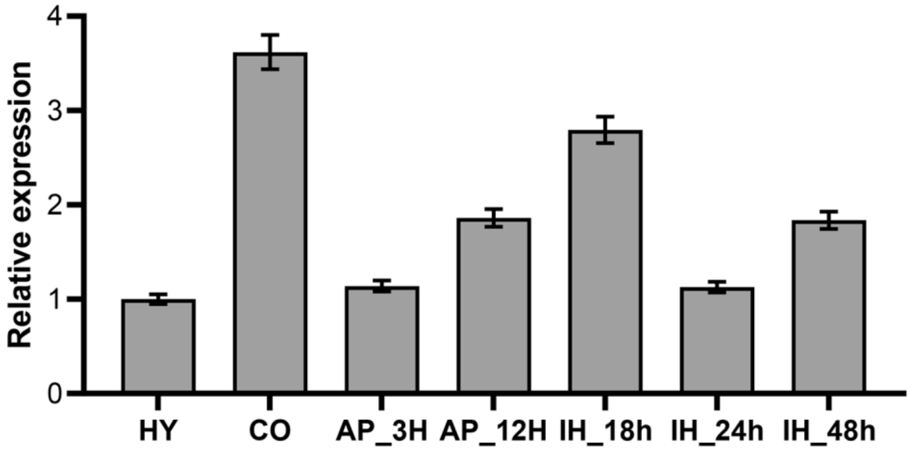

Supplementary Figure 2 | Expression of *RCN1* at different developmental and infection stages in *M. oryzae*. The actin gene *MoAct1* was used as an internal control. Expression level of *RCN1* in HY was set as 1. HY-hyphae; CO-conidia; AP-3h, appressorium at 3 h; AP-12h, appressorium at 12 h; IH-18h, infectious hyphae at 18 h; IH-24h, infectious hyphae at 24 h; IH-48h, infectious hyphae at 48 h.

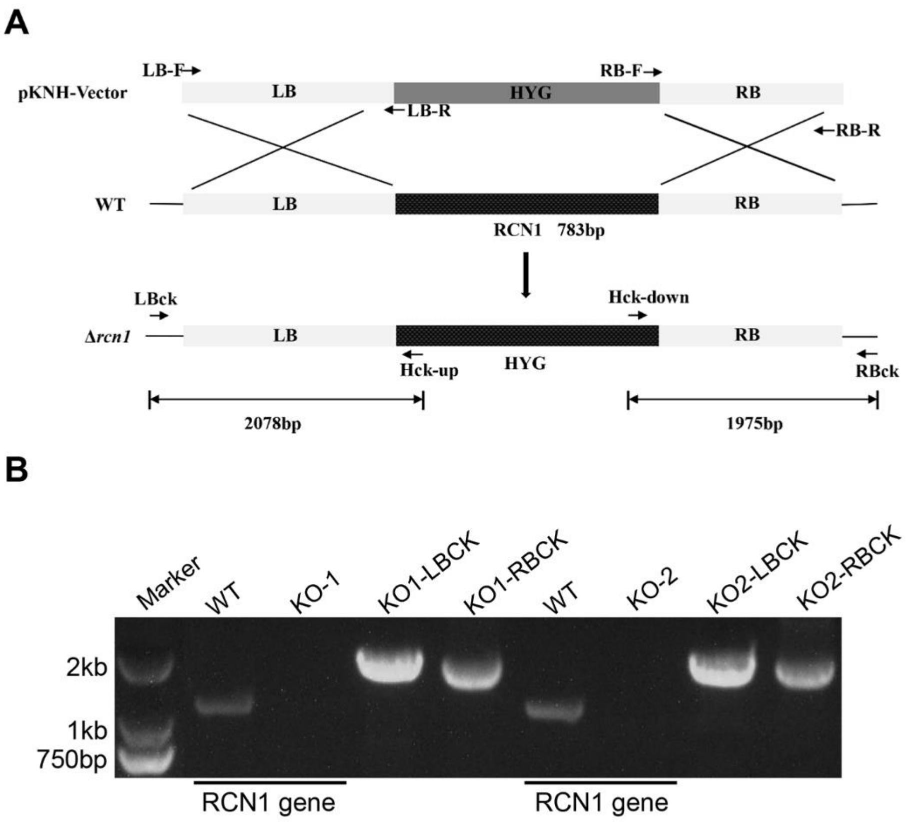

Supplementary Figure 3 | Replacement strategy and confirmation of *RCN1* deletion mutants. **(A)** Gene replacement of *RCN1* through a split-marker approach. White bars represent genomic regions upstream and downstream of the *PAL1* coding sequence that was amplified and fused to segments of the hygromycin phosphotransferase (*HYG*) cassette. **(B)** PCR verification of the deletion mutants. Flanking sequences besides the replacement fragment was amplified by using primer pairs of LCK/HCK-up and RCK/HCK-down. RT-PCR verification was performed by amplifying the *RCN1* gene in the transformants and the wild-type strain (WT).

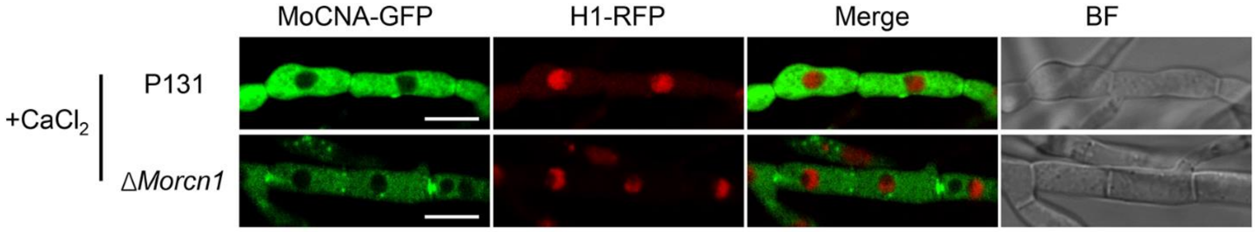

Supplementary Figure 4 | Subcellular localization of MoCNA in WT and Δ*Morcn1* mutants under high Ca^{2+} concentration level. MoCNA and H1-RFP were co-transformed into WT and Δ*Morcn1* mutant. The co-localization was observed under the confocal. Bar, 10 μm.

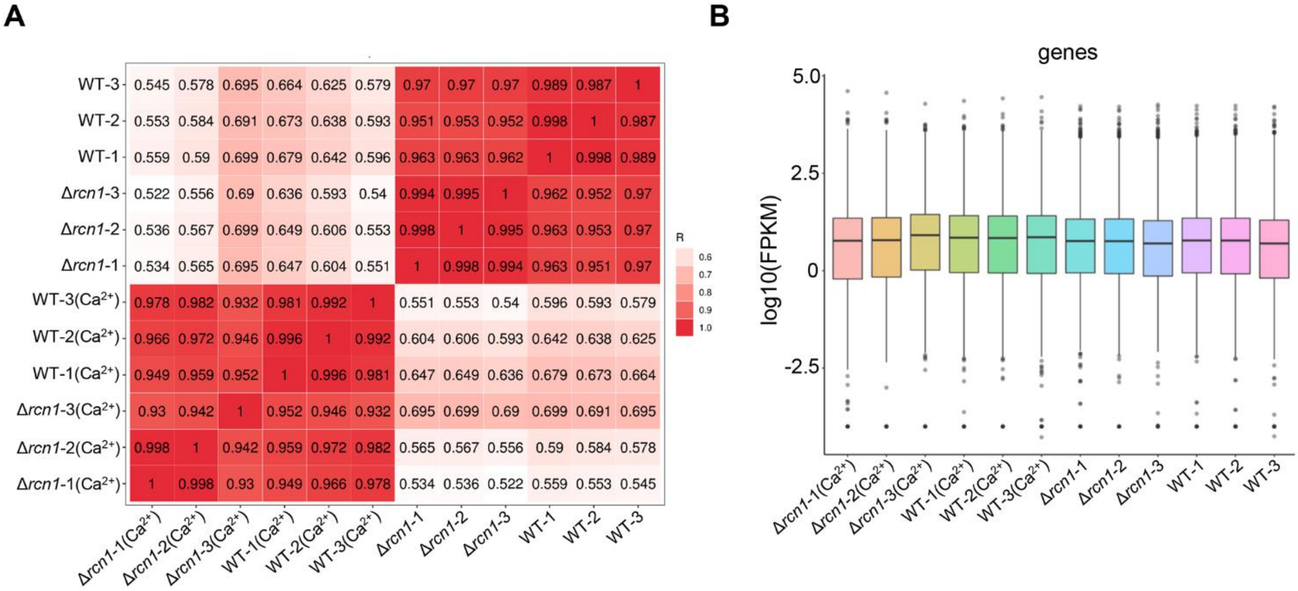

Supplementary Figure 5 | Correlation of different samples and normalization of RNA-seq data. **(A)** Pearson correlation between samples. **(B)** Boxplots showing the normalized values of gene expression detected in RNA-seq.
